# Austerity, Health and Public Safety in Low-Income Neighborhoods: Grassroots Responses to the Decline of Local Services in Southeast England

**DOI:** 10.1007/s11524-023-00807-x

**Published:** 2023-12-01

**Authors:** David M. Smith

**Affiliations:** School of Allied Health & Social Care, Anglia Ruskin University, Bishop Hall Lane, Chelmsford, CM1 1SQ Essex UK

**Keywords:** Austerity Policies, Deindustrialization, Health Access, Mental Health, Social Dislocation, Community Responses, Public Safety, Informal Policing

## Abstract

This article uses ethnographic and qualitative research to explore the health implications and social responses of a low-income neighborhood in Southeast England, to more than a decade of austerity policies and declining institutional and welfare support. Findings examine how cuts to public services and welfare programs alongside changes to the area’s social structure shape resident’s perceptions of health risks and threats. Residents pointed to poor levels of mental health that were exacerbated by financial insecurity, the closure of community facilities and difficulties accessing support and professional help. An increase in social disorder and sense of danger within the vicinity were attributed to changes in the area’s social composition and a reduction of policing in the neighborhood, which were an additional cause of anxiety for residents. Many people felt their neighborhood was treated inequitably with regard to law-and-order, health provision and other services designed to address health problems and risks and dangers in their social environment. This institutional vacuum generates unmet health needs facilitating informal practices and methods for managing health, such as through self-provision or using alternative, and more readily available, sources of medical advice and treatment. The demise of older forms of social control and surveillance that ran parallel with closure of the area’s communal spaces had been partly compensated by social media usage, while informal methods of policing were a growing presence in the neighborhood in reaction to rising lawlessness and the ineffectiveness of police and local authorities.

## Introduction

This article explores the social responses of one deprived neighborhood in Southeast England to the raft of austerity policies and welfare cuts enacted since the global recession of 2008 and continuing into the current “cost-of-living” crisis. The UK’s Conservative Coalition government (2010–2015) made reducing the budget deficit the key political priority with deep cuts to public spending and access to social benefits made increasingly conditional, part of a broader strategy to erode social protection and pass the economic burden of austerity onto lower-income groups [1]. Consequently, the costs have been borne unevenly both spatially and socially, with poorer areas and populations most adversely affected. Local authority (LA) budgets were cut by over half between 2010 and 2016 with social care, community and family centres, subsidised bus routes and housing programs particularly impacted. Deprived urban areas fared particularly badly with spending falling more in such areas fuelling inequality. Poverty in the UK grew by 2.2% annually between 2010 and 2020 to reach a near record of 36.3% on the Gini coefficient and with 22% of its population in poverty [2]. Despite the National Health Service (NHS) being protected from significant cuts, the public health impacts of austerity, welfare benefit caps and growing deprivation have been profound. Life expectancy is falling for the first time since the nineteenth century, and health inequalities have widened [3]. In 2018, the United Nations poverty envoy Philip Alston criticised the UK’s “punitive, mean-spirited and often callous” dismantling of its social safety net branding it a “social calamity and an economic disaster” [4].

Despite a body of knowledge detailing the regressive social and health impacts of austerity, there is little detailed ethnographic evidence into how austerity impacts spatially on health, or of the strategies that residents of low-income neighborhoods employ to alleviate those impacts [5, 6]. This paper will explore the lived experiences of those on the receiving end of austerity and the social dislocations it has entailed. It will do this firstly by examining how the micro-level impacts of cuts to public services and welfare benefits along with neighborhood-level demographic changes shape residents’ perceptions of proximate health risks and threats. Following this, it will explore the responses that emerge to mitigate potential health threats and dangers and which operate through localized information circuits and shared practices. Structural characteristics of neighborhoods, e.g. demographics, poverty levels, crime and social disorganisation, impact directly on health while influencing how health risks and threats are constructed and ranked [7]. While neighborhood mobilization in higher-income areas tend to centre on the preservation or enhancement of the areas’ positive features, in low-income areas, residents mobilize more in response to chronic, ongoing problems that directly impact their health and well-being [8].

## Study Setting

The study was conducted in a neighborhood in the Medway Towns conurbation 30 miles southeast of London with a population of 280,000. The area grew up around the Royal Dockyard at Chatham, which was established in the sixteenth century and once employed over 10,000 skilled workers and a further 10,000 in associated industries. Its closure in 1984 led to the loss of over 7000 jobs and thousands more in allied industries precipitating a period of economic and population decline [9]. While employment has since recovered, median earnings remain below the regional and national average with the transition from an industrial to a post-industrial labour market marked by a growth of low paid, flexible work and in-work poverty subsidised through welfare benefits [10].

Deindustrialization led not only to the collapse of the area’s economic base but also of the working-class cultures and systems of social production and reproduction characteristic of industrial capitalism [11]. Since its industrial demise extensive residential, retail and leisure development has taken place on its former dockyards and industrial land, reversing the fall in population, it receives the largest population outflow from London as the social cleansing of the capital has priced low- and middle-income households out of the capital’s housing market. This has also accelerated the dispersal of ethnic minorities away from London and other urban centres to make post-industrial Medway markedly more diverse as its ethnic minority population has grown from 6% to over 20% since 2001 [12].

Poverty and poor health have become increasingly concentrated spatially, and nearly one third of Medway’s neighborhoods are in the 10% most deprived nationally [13]. The study locale is one of its poorest with one third of its children in poverty compared to a local average of 18%. A third of its housing stock is “affordable” (i.e. low rent) almost double the area average. With unemployment only marginally higher than average, this is an area overwhelmingly comprised of the working poor. Over half its workers are in routine and manual work, average gross incomes are £10,000 lower than Medway’s average and one third of its working-households earn below £20,000 annually [14]. Despite having a younger age profile, it has the area’s highest proportion of long-term health problems or disabilities, poorer mental health and the lowest life expectancy [13].

The neighborhood has become more fragmented as the dominance of young families with children has been replaced by single-person “transient” renters living in Houses of Multiple Occupation (HMOs) who now form the largest residential group at 40%. This is followed by 25% families on low incomes and 15% lone-parent households. The area is also Medway’s most ethnically diverse with 20% of residents from black and minority groups and 10% “other-white” migrants (largely EU nationals). Indeed, EU8 (1) migrants aged under 30 were the largest group of new migrants settling in the neighborhood between 2004 and 2013 [15].

The neighborhood has long been stigmatized and has some of the region’s highest crime rates. The public nature of destitution, crime and anti-social behavior in low-income areas strengthens area-based stigma along class and racial lines and is closely correlated with perceptions of neighborhood safety and cohesion [16]. The most visible manifestation of austerity has been the large rise in local homelessness in the last decade with growing numbers of mostly men, begging and sleeping in the town’s high streets [17]. Findings examine how diminishing support from local government and state agencies and increasing destitution interact with neighborhood dynamics to shape perceptions of localized risks, and how residents mobilize to offset them.

## Methods

The study built on previous work undertaken in the area by the author to promote the integration of Roma migrants. Findings from the accompanying study indicated that despite specific issues related to their marginalized and migrant status, many of their main challenges were shared with other residents, e.g. low pay, long hours, debt and living in an area with a high concentration of social problems [18]. The closure or withdrawal of welfare services and amenities in recent years had exacerbated those problems especially the lack of mental health and family support services, precisely when demand for them was increasing.

Research took place between 2018 and 2019 and involved participant observation and discussions in various settings, e.g. at schools, resident’s homes, churches and social events. These were written up in a field diary which proved useful for focusing data collection, triangulating findings against documentary and interview data and adding depth to the analysis [19]. Four focus groups (*n* = 5 + 5 + 4 + 4) took place in a local school with residents consisting of 11 females and seven males aged 21–55. One limitation of the sampling methods is that they precluded access to a wider range of residents. Consequently, how the area’s elderly or various migrant populations’ experience and respond to the issues described in this article, or the extent they take part in the localized lines of action described, remains unanswered. Moreover, the sample size raises questions over whether participants’ experiences may be atypical and unrepresentative of the wider population. This indicates the need for long-term mixed method fieldwork in such neighborhoods to capture the diversity of experiences and responses to the “hollowing out” of public support and the health-related issues residents face.

Focus groups were organized by two female residents/community activists who were well-known to residents having lived in the area for many years and who publicized the study through neighborhood networks and at the local school. Those who expressed an interest were given an information sheet with details about the project and how to take part. Focus groups started by collecting basic demographic details (Table 1) and by asking residents to rank the neighborhood on a scale of 1–10. A topic guide was then used covering resident’s views on health in their area: what they considered the main health issues to be locally and their causes. Questions were asked to ascertain views on public services and how this compared with other neighborhoods: social support networks and experiences of local daily life. Focus groups lasted between 1½ and 2 h and were recorded verbatim before being transcribed. Interviews were also carried out in-person and by telephone with staff from schools and with representatives from the local authority, community organizations, charities and churches in the area.

**Table 1:** Focus group participants

	Gender	Age	Ethnicity	Status	Household members and ages	How do you rate the area from 1 (worst) to 10 (best)	Length of time lived in neighborhood
1	F	29	White British	Cohabiting	Partner (M) 31 Sons 10, 9	1	10 years
2	F	35	White British	Married	Husband 34 Daughters 17, 11, 10 Daughter 3	4	8 years
3	M	27	Mixed/Black British	Cohabiting	Partner (F) 26 Son 2	3	3 years
4	F	36	White British	Married	Husband 35 Sons 15, 10 Daughter 12	4	< 1 year
5	M	36	EU National	Single	N/A	3	5 years
6	M	30	EU National	Cohabiting	Partner (F) 30 Son 2	2	3 years
7	F	44	White British	Divorced	Sons 21, 11	5	18 months
8	M	21	White British	Living with family	Mother 39 Brother 17	4	20 years
9	F	32	Black British	Separated	Son 3 Daughter 18 months	4	2 years
10	F	31	British/Gypsy	Married	Husband 34 Sons 14, 13 Daughter 10	7	4 years
11	M	55	British/Gypsy	Married	Wife 54	5	29 years
12	F	32	White British	Divorced	Daughter 11, 9, 2 Sons 5, 4	5	13 years
13	F	42	White British	Married	Husband 35 Son 13 Daughters 11, 9	2	8 years
14	M	46	White British	Married	Wife 35	1	2 years
15	F	39	EU National	Married	Husband 43 Daughters 15, 13	3	4 years
16	F	41	White British	Divorced	Sons 16, 14, 8	2	18 years
17	M	30	EU National	Cohabiting	Partner 27	3	2 years
18	F	24	White British	Single	Sons 4, 2	3	5 years

Transcripts were read through after each focus group and manually coded by themes. Emergent themes were then incorporated into the topic guide and explored further in subsequent focus groups through constant comparative assessment, whereby coding and analysis take place concurrently. Using this approach, the interplay between themes and categories became increasingly refined and focused. The process is similar to grounded theory where theory is developed from data, though here it is applied to provide an explanatory account [20]. Participants provided written consent before data collection and were offered a £20 gift voucher for taking part after the focus group. The study received ethical approval from Anglia Ruskin University’s Faculty of Health Social Care and Education Research Ethics Panel.

## Results

Analysis of results indicated the spatial impacts of austerity and the decline, or withdrawal, of local services worsened residents’ economic and social precarity, with negative impacts particularly on mental health. Residents pointed to geographic disparities with their neighborhood treated inequitably in health provision, policing and other services compared with other local areas. This institutional vacuum resulted in unmet needs and encouraged alternative grassroots practices for managing health and public safety in localized networks structured around relations of trust and mistrust.

## Disparities between Health Needs and the Provision of Health and Community Services

Residents highlighted a spatial patterning of poor mental health, which is seen as the most serious health problem locally with its prevalence attributed to high levels of poverty and economic insecurity. Over time, this has a corrosive impact psychologically, with one participant noting, “when issues are about money indoors that can bring on anxiety and how the mind is functioning to keep the household going every day it’s a constant worry”. These stressors were intensified by debt and limited access to financial advice. Outside of household pressures, low-income neighborhoods bore the brunt of local service cuts often with far-reaching implications. Closure of the local family centre was viewed as a catalyst for the corrosion of community support. One resident recalled “when we had the family-services up and running here this community was a damn sight better”, while others described how the centre had provided a vital site for adults and children to socialize and access services like playgroups and parenting classes,

In the summer holidays we’d have days when none of us had any money, we’d all go in the community-centre, the kids would eat toast, a little bit of juice. We all helped each other out and now there’s nothing.

Participants argued that its closure had a serious impact on opportunities for social mixing and for tapping into local informal support mechanisms thereby increasing social isolation. One resident related the centre’s closure to the seemingly worsening mental health of residents, commenting that “more people suffer with mental-health in this area and when you have that support for families you can see now what happens when it’s taken away”. As discussed further, loss of the centre would have wider ramifications on social relations and how community information and local threats were conveyed locally.

Deprived areas tend to be doubly disadvantaged in health terms since they are served by fewer GPs and health facilities compared to wealthier areas [21]. The paucity of local health facilities and economic constraints was a serious obstacle to accessing health care, highlighting the inverse relationship between health needs and its geographic provision. One mother highlighted the practical difficulties this caused when accessing health care.

You can’t walk there and back with your children, you haven’t got money for buses and taxis we haven’t got GP surgeries, we haven’t got walk-in centres but we’re the poorest town.

The withdrawal of community support and poor health access worsens mental and physical health problems, concentrating them spatially within certain neighborhoods and households as one participant observed, “it’s not one house, there could be multiple people in that one house that’s got mental health problems”. Another, discussing the lack of support for her teenage daughter, starkly illustrated how poor mental health accumulates within households without adequate support.

Some days I have such a bad headache and I’m puking up with the stress because I’m thinking where do I take her what do I do with her? So, my health suffers because I’ve nowhere to go [for help].

This section has outlined the uneven spatial outcomes of austerity measures and some of their health impacts on the people most adversely impacted by them. The next section continues this theme by exploring how the insecurities and anxieties that these policy-induced changes entail are further aggravated by the increasing spatial concentration of social problems, destitution and lawlessness.

## Fragmentation, Policing and Neighborhood Risks

The sense of threat locally stems partly from the demographic shift from homogeneity, which promotes shared values, practices and informal social control towards heterogeneity amidst uniform poverty. Some participants argue this has encouraged a breakdown of social order as one long-term resident observed, previously “everyone knew everyone and now they don’t we’ve got more street-crime, robberies, drugs, prostitution”. Secondly, was the local impact of police funding cuts and lack of neighborhood policing with participants highly critical of the poor responses to crime. Cuts to Police Community Support Officers (PCSOs) were felt disproportionately in the neighborhood, with one participant noting “we’ve got no police at all. We’ve haven’t got PCSO officers” (2). The loss of PCSOs severed a key link between the police and community as one participant recalled,

…when we used to have community-police that used to go in and stop by for a chat see what was going on, we’ve got none of that no more.

Many reported feeling “abandoned”, arguing that the reduction of policing and disengagement of state agencies from the area had encouraged crime and disorder, as highlighted by the following participant.

what’s happening is that if you leave an entire community to run and police itself, you’re going to have anarchy and that’s what you’ve got round here now its anarchy.

Despite experiencing some of the highest crime, locally participants also felt the area fared poorly in crime surveillance and prevention compared to neighboring areas. Poor street lighting and a lack of CCTV were a major concern as the following participant highlighted “if this community’s crime rate is so high then why are we the only estate that has got no CCTV? Everywhere else has it”. Following a spate of assaults in a nearby alleyway, participants argued that the reduction of services to maintain the neighborhood’s environment also created situations where personal safety was compromised. This caused resident’s considerable anxiety with many feeling that their concerns were not prioritized by local officials.

I spoke to [local councillor] about getting security or cameras, lights anything, block the alleyway off do something to stop it happening again. But then obviously everyone wants funds and it just isn’t there. Not for our kids anyway.

Risks emanating from within the neighborhood’s deteriorating public spaces, restricted social activities especially for women and children. Green zones are positive contributors to health and well-being, narrowing health disparities and strengthening neighborhood identities [22]. However, the local park was considered dangerous and out-of-bounds, as noted by the following parent “…where are the kids going to play? My kids aren’t going up the park because I don’t know what nonces are walking past” (3). The local authority’s failure to address residents’ concerns and the widespread sense of abandonment would create contexts where alternative strategies develop. These grassroots responses function as informal, and partial, solutions to the intensification of spatially bound social and health risks on one hand, and to the decline or withdrawal of public mechanisms to manage those risks on the other.

## Resident Strategies for Managing Inadequate Health and Community Services

The following sections outline some of the micro-level responses that have emerged in response to the structural forces and government policies that have decimated public support and services in poor neighborhoods throughout the UK [23]. Frequently, the coping mechanisms participants employ to manage health issues impacting themselves and family members would incur additional costs for families already struggling financially. Despite experiencing severe economic hardships, one mother explained how she was paying for home tuition for her young son who has severe anxiety and is unable to attend school. Home tuition is not funded by the local authority, and her son is on the waiting list for support amidst a national crisis in children’s mental health care [24].

I’ve got to fund that. I’m paying myself because obviously I need my child to learn. I’ll quite happily pay that but my point as a parent is that my four-year-old should not be suffering like this.

Others recalled friends and family members having to use drastic measures after unsuccessful efforts to access mental health support.

She was ringing mental-health. Nobody helped her. She phoned the doctor’s surgery. In the end she said “I’m telling you now if somebody don’t come and see me, I’m going to fucking kill myself” and put the phone down.

Given the lack of local health facilities, many residents used the local pharmacy when they or their children became sick. One participant noted this was a quicker and cheaper method of treating his tooth infection compared to visiting the dentist,

I told him [pharmacist] “I’ve got a toothache and infection in my mouth” paid £10 and he gave me a prescription which is brilliant. You can go there for anything.

While this meant that symptoms could be assessed quicker than through conventional routes, this could also incur additional costs since prescriptions are usually free for under 16s. Nevertheless, the time and expense of attending the nearest medical centres meant the chemist was often the preferred option.

I don’t take my kids to the doctors I take them to the chemists and I’ll pay for their prescriptions but I know that we shouldn’t have to pay for children’s prescriptions.

Neighborhoods are not bounded spatial entities but interrelated elements in the hierarchical positioning of cities within global networks of unequal economic, political and cultural power—and of particular places and institutions within those cities [25, 26]. While there has been a decoupling of neighborhoods like the one described here from state institutions, many aspects of residents’ lives are intensely supervised and managed by professionals employed by the state or associated organizations [27]. Rarely are these interactions or their outcomes positive or enabling and are more frequently experienced as oppressive and contradictory. One participant illustrated this, recalling a discussion with her housing officer.

She [housing-officer] keeps going “well you’ve got to pay housing, your house first blah, blah, blah” and I was thinking, hold on a minute if I don’t feed my kids, I’m going to have social services at my door. What would you want?

The next section discusses how information concerning local threats are communicated and acted upon by residents at the neighborhood level. These represent collective attempts to maintain control over local public space against a background of depleted community resources and insufficient policing.

## Community Surveillance, Informal Policing and Neighborhood Risks

The networks that disseminate local knowledge, gossip and informal surveillance in the neighborhood were transformed by the loss of community spaces. One participant recalled that when there were more sites for social interaction, “there wasn’t one thing that we didn’t know about under the old system. We knew about everything.” Social media has partly substituted for the decline of physical spaces allowing for the rapid spread of local news concerning localized dangers, such as the capture of alleged paedophiles by self-styled “paedophile hunters”.

It wasn’t common knowledge like it is now. Thank God there’s the internet and Facebook because otherwise we’d have never known what was going on.

Fears that child abusers lived in the vicinity and the perceived absence of official action to either tackle such threats, or to share information with residents, were common as one participant observed,

They’ve lived here forever. It’s not like someone’s moved in and you didn’t know, some of them probably lived here longer than we have.

These deeply held concerns had two main outcomes that shaped community dynamics and the nature of the response. First, it generated mistrust and suspicion that largely confined social support within networks based on long-standing ties of locality, friendship and kinship. Second, it strengthened the belief that the area was detached from, and ignored by, state institutions designed to protect them as the following resident argued,

Nobody knew, why hasn’t the police been telling us? We’ve got babies go to these schools and play out on the streets and we don’t know about it.

The most notable illustration of the minimal confidence in official agencies was the emergence of informal and unofficial community surveillance and policing. This was conducted largely by the neighborhood’s males. While the public presence of large groups of young men in poor localities is often regarded as a sign of danger and disorder by outsiders, the following resident told a different story.

That’s why you have the big groups of men and big groups of boys. Don’t want them here [paedophiles and drug-dealers] and the police ain’t doing nothing. I don’t know if they don’t care or they’re scared but nothing is being done.

Many of the women particularly reiterated that the deficit of law-and-order meant public safety was provided from within the neighborhood not from official agencies: one woman commented “what’s happening here the only reason we’re safe is because the men on this estate are policing it for us”. Another argued that informal security was effective in deterring social deviants and wresting control of public space by indicating to potential wrong-doers “once you’re more visible in our community and then they [criminals etc.] know you lot ain’t tolerating that”.

The dilemma for such grassroots responses to perceived local dangers is that ultimately, they can fuel the disorder and insecurity that they were designed to address. The line between informal policing and vigilantism is a thin one especially when the latter is perceived as the only available recourse against undesirable locals. Likewise, the balance between maintaining a visible presence to deter criminal activity and taking reprisals against transgressors can tip towards the latter, when state agencies are seen as failing to execute their duties. One participant argued that.

It won’t be long before houses get burnt down and all of that because you’re going to find that people go “Do you know what, you’re [police, social-services] taking the piss we’ll get them out ourselves”.

Residents’ frustration at feeling spatially excluded from protection by the state and its agencies and perceived inaction against local criminals and social deviants therefore meant the threat of violent reprisals and a further breakdown of social order remained a constant possibility.

## Discussion

Findings reinforce previous research on the uneven spatial impacts and calamitous social and health outcomes of austerity, welfare retrenchment and the growing detachment of low-income neighborhoods from systems and institutions of local governance [1, 3–5]. In this sense, austerity not only has spatial *effects* but also is itself akin to a spatial *force* that causes degradation to the social infrastructures of coexistence, diminishing their potential to provide a dignified life and modicum of social and economic security [28]. The ethnographic and qualitative approach in this article illustrates how these forces are experienced spatially through its insights into their corrosive impacts at community and household levels and the highly localized constraints they generate, e.g. in accessing public spaces, health, support services and policing. Results highlight some of the strategies residents use to circumvent non-existent or extremely limited public provision and illustrate how the decimation of local services, policing cuts and high levels of street crime generates spatialized fears and insecurities that were articulated as a loss of legitimacy for the state and a lack of confidence in its institutions. Subsequently, there was growing support for grassroots forms of mobilisation like informal policing and public vigilantism—typically seen in developing nations where policing is severely under-resourced and ineffective [29, 30]. Winlow et al. are critical of the “appreciative” modes of enquiry that compete with dominant neoliberal representations of the poor. While the latter demonize and pathologize, the former overemphasizes the positive aspects of poverty: the solidarity, resilience and stoicism of the poor while downplaying social breakdown, rage and desperation [31]. Likewise, the neighborhood and household responses to the “hollowing out” of institutional support reported here were rooted not so much in collective resilience as desperation that health, welfare and policing have declined to the extent that they can no longer be relied upon and in deep anxieties over social disorder, public safety and the extent of poor mental health, poverty and deprivation locally. Changing demography was also central to understanding the weakening of social order with the transition from social homogeneity towards increasing heterogeneity, fostering a heightened sensitivity to dangers within the neighborhood and strong perceptions of danger and insecurity with impacts on psychological health and well-being [32]. Mistrust stems from the interaction between people and place, while the structural properties of disadvantaged neighborhoods can intensify resident’s disposition for mistrust [33]. An increasingly diverse low-income population did not diminish sociability and mobilization, but structured it around relations of trust originating in shared backgrounds, personal loyalties and close knowledge of individual histories [34].

The economic and social precarity framing the participants’ lives has worsened considerably since fieldwork, with the “cost-of-living” crisis disproportionately impacting those on low incomes and accompanied by sharp rises in poverty, debt and use of charities and foodbanks [35]. It also has a clear geographical pattern with the UK’s poorest cities, towns and areas being most adversely impacted by falling living standards [36]. The current government’s “Levelling Up” policy agenda to reduce the UK’s entrenched regional and spatial inequalities has released £3.8bn for areas to bid for cultural, town-centre and regeneration projects of which Medway received £14.4m in the first round. However, it is hard to see what the three projects it funded, to transform the town into a leading “creative destination”, will do in the short-to-medium term for its poorest residents, and with a £35.5m real-term loss in government funds since 2018 Medway, like the rest of the country, has lost far more local authority funding since 2018 than it has gained through Levelling Up [37]. Findings indicate the urgent need for a broad programme of national reform to reverse the stark social and economic divisions and their spatialized outcomes currently afflicting many of the UK’s former industrial towns. In particular, area-based interventions and targeted health equity actions are critically needed to address the growing social and health inequalities in those neighborhoods left to fend for themselves.

## Data Availability

Data generated and analyzed for the current study are not publicly available.
